# Targeting the Salience Network: A Mini-Review on a Novel Neuromodulation Approach for Treating Alcohol Use Disorder

**DOI:** 10.3389/fpsyt.2022.893833

**Published:** 2022-05-17

**Authors:** Claudia B. Padula, Lea-Tereza Tenekedjieva, Daniel M. McCalley, Hanaa Al-Dasouqi, Colleen A. Hanlon, Leanne M. Williams, F. Andrew Kozel, Brian Knutson, Timothy C. Durazzo, Jerome A. Yesavage, Michelle R. Madore

**Affiliations:** ^1^Mental Illness Research Education and Clinical Center, VA Palo Alto Health Care System, Palo Alto, CA, United States; ^2^Department of Psychiatry and Behavioral Sciences, Stanford University School of Medicine, Stanford, CA, United States; ^3^Department of Psychiatry and Behavioral Sciences, Medical University of South Carolina, Charleston, SC, United States; ^4^Department of Neurosciences, Medical University of South Carolina, Charleston, SC, United States; ^5^Department of Cancer Biology, Wake Forest School of Medicine, Winston-Salem, NC, United States; ^6^Department of Behavioral Sciences and Social Medicine, Florida State University College of Medicine, Florida State University, Tallahassee, FL, United States; ^7^Department of Psychology, Stanford University, Stanford, CA, United States

**Keywords:** alcohol use disorder, neuromodulation, transcranial magnetic stimulation, treatment, salience network, neurocircuitry

## Abstract

Alcohol use disorder (AUD) continues to be challenging to treat despite the best available interventions, with two-thirds of individuals going on to relapse by 1 year after treatment. Recent advances in the brain-based conceptual framework of addiction have allowed the field to pivot into a neuromodulation approach to intervention for these devastative disorders. Small trials of repetitive transcranial magnetic stimulation (rTMS) have used protocols developed for other psychiatric conditions and applied them to those with addiction with modest efficacy. Recent evidence suggests that a TMS approach focused on modulating the salience network (SN), a circuit at the crossroads of large-scale networks associated with AUD, may be a fruitful therapeutic strategy. The anterior insula or dorsal anterior cingulate cortex may be particularly effective stimulation sites given emerging evidence of their roles in processes associated with relapse.

## Introduction

Alcohol use disorder (AUD) is the most prevalent substance use disorder (1), imposes the greatest burden of illness (2), and alcohol-induced deaths in the United States are currently on the rise (3). AUD is associated with poor medical, psychological, and social outcomes, such as adverse overall physical health, neuropsychological deficits, psychiatric comorbidities, homelessness, unemployment, and relationship dysfunction (4–6), which contribute to a subsequent poor quality of life for individuals suffering from this devastative disorder. The estimated costs to the American economy were $223.5 billion in 2006 for excessive alcohol drinking (7). Current evidence-based interventions, be it psychotherapy or pharmacological, still result in approximately two-thirds of individuals relapsing by 6-months post-treatment (8). Emerging research into the key brain-based factors that contribute to relapse may reveal novel targets for prediction, intervention, and relapse prevention. To this end, we aim to briefly review the neurocircuitry of addiction, the role of the salience network (SN) in treatment outcomes, the latest trends in neuromodulation for psychiatric disorders, and conclude with a potential avenue for advancing brain-based interventions for AUD.

## Fundamental Conceptualization of Addiction: A Brain-Based Condition

### Neurocircuitry of Addiction

Converging lines of evidence from preclinical and human research have resulted in the empirically driven, brain-based conceptual framework of addiction. Animal and human models of addiction have allowed for a sophisticated interrogation of the neurocircuitry that underlies addictive behaviors. Koob and colleagues describe the dynamic allostatic process of the addiction cycle in three stages: (1) binge and intoxication, (2) withdrawal and negative affect, and (3) preoccupation and anticipation stage (9, 10). During the binge and intoxication stages, the reward system is initially hyperactive, specifically the ventral tegmentum and the nucleus accumbens, and following chronic alcohol use, a homeostatic shift to hypoactivation occurs. During withdrawal and negative affect, there is a focus on reduced experiences of reward for conventional stimuli and increases in negative affect. These psychological experiences are coupled with changes in the striatum, extended amygdala, and insula functioning. Finally, the third stage is influenced by stress, increased disinhibition, and leads to preoccupation and anticipation of reward, including craving, which increases relapse risk (11, 12). A core part of this model is the shift from impulsivity fueling early stages of addiction to compulsivity fueling later stages of addiction (including relapse). This co-occurs with a shift from positive reinforcement mechanisms to negative reinforcement mechanisms, which can drive motivated behaviors. At the crossroads of this transition is the SN (13).

### The Salience Network

The SN is a multifunctional, intrinsically connected, large-scale neural circuit implicated in several psychiatric conditions, such as addiction. Specifically, the SN is associated with the detection of salient changes in the environment, both interoceptive and external, and signals the need for cognitive control (14). Critical cortical nodes of the SN include the anterior insula (AIns) and the dorsal anterior cingulate cortex (dACC) (14–16). Additionally, these core nodes functionally connect with subregions of the prefrontal cortex (PFC) inferior parietal lobule (IPL) (14, 16–18) and downstream, subcortical regions of the extended amygdala, ventral striatum, and substantia nigra/ventral tegmental area (19, 20). Although intrinsic connectivity of the SN is most often reported during states of rest in humans, SN function is also interrogated during active tasks involving cognition, action, and emotion (19).

Recent work confirms these SN nodes are paralleled in preclinical models (21). In humans, the AIns, dACC, and dorsolateral PFC (dlPFC) co-activate in response to tasks of cognitive demand, cognitive control, decision-making, and environmental uncertainty. This co-activation has previously been implicated in negative mood states (17, 22–24), and synchronous activation has been shown to increase with task difficulty and stimulus ambiguity. This finding, of task difficulty-dependent, increased activation, suggests that the dACC and AIns both play an integral role in cognition by filtering and integrating internal and external stimuli during a variety of cognitive tasks (25).

The insular cortex is involved in various cognitive and affective processes, such as responding to internal and external emotionally salient stimuli, decision-making, threat recognition, and conscious urges (26). The insula is also heavily implicated in interoception, which involves integrating a wide array of somatic physiological conditions to maintain homeostasis (27, 28). The role of the AIns in the SN includes bottom-up detection of salient stimuli *via* integration across perceptual modalities (29–33). Specifically, the AIns plays a key role in externally orienting attention and internally orienting self-related cognitions through engagement with default mode and executive networks (14, 34–37).

Conversely, the dACC plays a crucial role in initiation, motivation, and goal-directed behaviors (38–41), with key projections that influence motor responses (42–44) and interactions with other large-scale networks that have a major role in motor/behavioral selection (25). In addition to co-activation with separate functional networks, the dACC also possesses extensive cortico-cortical connections within the PFC, including the dorsolateral prefrontal cortex (dlPFC) and premotor regions, making it critical for learning and behavior (45). Taken together, the fundamental nodes of the SN—the AIns and dACC—are implicated in the processing and synthesis of several complex human experiences, such as cognition, action, and emotion (19). These critical functions are central to the development and maintenance of addiction (10).

### SN in AUD: Intersection With Neurocircuitry of Addiction

Regardless of clinical phenotype, the AIns and dACC are consistently implicated in the development and persistence of multiple psychiatric disorders, such as addictive disorders, suggesting they are critical for psychological well-being and adaptive functioning (46–48). For addiction specifically, the SN may interact and influence incentive salience (49, 50), negative affect (51–55), and executive function (56–60) networks, which are core neurocircuitry underlying AUD (61, 62). The Competing Neurobehavioral Decision System theory, alongside the Impaired Response Inhibition and Salience Attribution models, among others, are established conceptual frameworks of addiction that unite both behavioral and neurobiological systems involved in AUD (63). These models specify that the cortico-striatal circuits involved in processing salient internal or external stimuli, as well as cognitive decision-making, are compromised in AUD. Preclinical literature has identified regions, such as the dACC, insula, and striatum, as targets that are causally linked to alcohol-seeking behaviors (64). In humans, SN abnormalities contribute to difficulties with impulsivity, compulsivity, and executive dysfunction (65, 66), and an increased relapse risk in AUD (4).

The first study to suggest the AIns may have a critical role in the addiction cycle was by Naqvi et al. (67), showing that structural damage to the insula disrupted cigarette consumption. Following a right or left insula lesion, individuals demonstrated rapid and extended smoking cessation, had fewer conscious smoking urges during abstinence (67), and were five times more likely to quit smoking compared with people with no insula lesion (68). Structural damage to the insula has also been shown to decrease the occurrence and severity of nicotine withdrawal symptoms (69), and smoking cessation difficulty (68). Taken together, these fundamental lesion studies highlight the role of the insula in withdrawal and relapse.

Dysfunction of the dACC has also been intensively described as having a role in psychiatric conditions, such as the development and maintenance of AUD. For example, prior studies have demonstrated that reduced dACC activation and compromised connectivity of the SN nodes are associated with greater decision-making latency in AUD (70, 71). Similarly, in individuals who reported binge drinking, acute alcohol consumption caused blunted functional connectivity between the bilateral AIns and the ACC (72). Several groups have demonstrated that such neurobiological abnormalities of the SN are related to the inability to restrain subjective urges (71) and evaluate emotionally salient stimuli (72) in AUD, further supporting that dysregulation of the SN in AUD (63), across resting state, social and emotional processing, and inhibitory control tasks, such as specific reductions in blood flow (73). Acute alcohol consumption significantly attenuates bilateral anterior insula activation to emotional face cues relative to neutral faces and is exacerbated by the level of response to alcohol, which increases the risk for AUD development (74, 75). SN dysfunction, such as structural and metabolic abnormalities (4, 5, 76) and reduced functional connectivity among nodes of the SN, is also predictive of future relapse in AUD (77, 78).

Similar evidence also points to the insula and the dACC playing major roles in reactivity to alcohol cues. A systematic review of over 100 task-related imaging studies by Zilverstand et al. revealed hyper-activation and hyper-connectivity during substance cue exposure, but blunted activation and reduced connectivity during all other tasks, such as cognitive control, non-substance reward, and social/emotional tasks (63). Other cue reactivity studies have found that neural activation in the insular cortex and the ventral striatum can be used to differentiate between heavy and light alcohol drinkers, with heavy drinkers having a higher activation in those regions in response to alcohol cues (79). This differentiation of neural activation between levels of alcohol use may also provide insights into who is at the highest risk of relapse (26). For example, Kohno and colleagues reported that individuals who did not complete AUD treatment showed increased resting-state connectivity between the striatum and the insula, demonstrating that SN dysfunction could be predictive of future drinking (77).

In summary, evidence suggests that insula and dACC activation and connectivity to other key nodes of the SN and how they relate to the neurocircuitry of addiction are highly relevant to the development and maintenance of AUD. One approach to improving treatment outcomes for these individuals may be to directly target SN function through novel therapeutic techniques that have demonstrated efficacy in other psychiatric conditions.

## Research Gaps: Advancing Treatment for AUD

Among the different treatment options available for AUD, inpatient detoxification for alcohol appears to be the most frequently utilized (80). Residential treatment programs typically apply pharmacotherapies and/or behavioral interventions, such as cognitive-behavioral therapy, group-based peer support, and relapse prevention strategies. However, even with extensive, residential treatment, relapse rates remain high. One potential limitation of existing interventions is that they modify behaviors globally with indirect effects on the brain. Non-invasive neuromodulation techniques demonstrate promise by modifying specific and selective neural targets shown to be associated with symptoms. The current modest efficacy of evidence-based interventions, combined with increasing rates of alcohol-related deaths, makes the development of new brain-based therapeutics a high priority.

Transcranial magnetic stimulation (TMS) is a brain modulation technique that involves the use of different frequencies and patterns of stimulation to generate an electromagnetic field to depolarize neurons and influence cortical excitability. Apart from having FDA clearance for treatment, several resources describe guidelines for treatment and safety protocols (81–86). Extensive research supports the clinical efficacy of TMS for psychiatric disorders, most commonly for major depressive disorder (MDD), after FDA approval in 2008 (81). Since 2008, TMS has received FDA clearance for the obsessive-compulsive disorder (OCD) and smoking cessation (87, 88). Researchers have aimed to expand TMS indications for comorbidities associated with MDD, such as OCD, bipolar disorder, PTSD, and substance use disorder (83, 84). Additionally, researchers have tested altering the standard treatment protocol (89) or using high-efficiency forms of TMS, such as intermittent Theta Burst Stimulation (iTBS) (90) in hopes of increasing efficacy and/or decreasing overall treatment time.

To date, treatment approaches for TMS in AUD have predominately targeted two brain regions: the dlPFC, and the medial prefrontal cortex (mPFC). While meta-analytic studies are difficult to utilize given the inconsistency in the treatment parameters used (e.g., as shown in Ref. (91), a few brief trends have emerged: (1) 10 Hz left or right-sided, dlPFC protocol for the treatment of AUDs is generally helpful in reducing craving (92, 93); (2) the right-sided dlPFC has variable results which may or may not be related to the frequency at which treatment is delivered (92, 94); (3) mPFC stimulation consistently reduce brain reactivity to alcohol cues (95–97), and may reduce alcohol use post-treatment (98, 99); and (4) regardless of stimulation site and chosen TMS parameters, applying 10+ sessions of TMS appears to consistently decrease alcohol craving (94, 98, 100, 101). While results are promising, it is notable that many of these studies focus on craving and have not directly reduced alcohol consumption or relapse risk (92, 94–97, 100–106). While the mixed results within the field may be due to differences in parameter application (frequency, strength of stimulation, and number of sessions), another limitation may be that protocols were only able to stimulate the outermost cortex, rather than deeper nodes within the SN.

Recent technological advances in TMS coil design have made it possible for TMS-induced electric fields to penetrate deeper into the brain, modulating areas, such as the dACC and AIns in addition to superficial cortical areas (107). Recent studies indicate that these newly developed H-coils tend to provide both a broader area of stimulation and increased depth as compared with the Figure-of-8 coil [see Tendler et al. (107) for a review of the various H-coil designs and exact cortical targets]. Furthermore, the design of the H-coils can provide simultaneous activation of both the left and right lateral and medial prefrontal cortices depending on the specific coil and the treatment parameters used (107). Although this design stimulates both hemispheres, there is evidence to suggest that it stimulates the left hemisphere more than the right (108, 109). Given that the electromagnetic fields delivered by TMS decay exponentially with distance (110, 111), specific confirmation is needed to determine if these deep TMS devices can stimulate subcortical regions, such as the AIns and the dACC. This is of particular relevance in AUD, wherein alcohol is known to induce widespread cortical atrophy (112). That said, a recent study investigating the distance from the scalp to the cortex at the dlPFC and mPFC among a sample of individuals with AUD and healthy controls did not find a significant difference between the groups (113).

Similar to the mood disorders literature, targeting may be critical in addition to overall dosage (i.e., the total number of pulses administered during a treatment course) when considering how best to achieve downstream network effects (114). Table 1 summarizes all TMS studies to date in AUD, which clearly emphasizes that the field of targeting subcortical nodes within the SN is in its infancy. Among the sparse, existing literature, evidence suggests that cue-induced craving may be better modulated by targeting inferior structures, such as the ACC, the insula, or the mPFC (99, 122). However, the literature on how to best modulate insular and cingulate activity also provides disparate information. Results from Perini et al. suggest that insular stimulation made no difference in resting-state connectivity or craving in treatment vs. sham groups (121) for AUD. These results are particularly important to consider given the involvement of the insula in AUD circuitry (26). Harel and colleagues recently reported that an H7 stimulation protocol targeting the dACC resulted in changes in functional connectivity *and* fewer heavy drinking days in the active condition compared with the sham condition (98). While these early works have produced mixed results, the converging preclinical and clinical evidence regarding the centrality of SN nodes in the development and maintenance of AUD warrants further investigation.

**Table 1:** Summary of all transcranial magnetic stimulation (TMS) studies in alcohol use disorder (AUD) to date.

**Ref**	**N (Active, Sham)**	**rTMS parameters**					**Outcome measure**				**Findings**	**Blind**	**Active sham** **control**
		**Site**	**Hz**	**%MT**	**Sessions; Duration**	**Pulses/** **Session**	**Drinking Behavior**	**Craving**	**Brain/ Biology**	**Other Bx**			
Mishra et al. (93)	45 (30,15)	R.dlPFC	10	110	10 1 month	1,000	1-mo relapse	ACQ-NOW	N/A	N/A	↓ in craving; relapse 14% active, 33% sham	S	Y
De Ridder et al. (115)	1	dACC	1	50	1 1 day	600	N/A	VAS	BOLD	N/A	↓ in craving ↓ BOLD in dACC and PCC	N/A	N/A
Höppner et al. (102)	19 (10,9)	L.dlPFC	20	90	10 10 days	10,000	N/A	OCDS	N/A	AB	No diff in craving/dep sx ↑ in AB effect to alc-stim	S	N
Herremans et al. (103)	31 (15,16)	R.dlPFC	20	110	1 1 day	1,560	N/A	OCDS	N/A	N/A	No diff in craving	S	N
Herremans et al. (104)	29 (29,29)	R.dlPFC	20	110	1 2 days	1,560	N/A	OCDS	N/A	Go-NoGo	No difference in craving ↓ IIRTV of Go-NoGo	S	N
Ceccanti et al. (99)	18 (9,9)	dmPFC	20	120	10 10 days	1,000	TLFB	VAS	cortisolemia, prolactinemia	N/A	↓ craving ↓ # drinks per day/max ↓ cortisol and prolactin	D	Y
Girardi et al. (100)	10	L.dlPFC	20	120	20 1 month	2,200	N/A	OCDS	N/A	N/A	↓ craving/depressive sx	N/A	N/A
Herremans et al. (94)	26 (13,13)	R.dlPFC	20	110	15 4 days	1,560	N/A	AUQ, OCDS	BOLD	N/A	↓ craving, not cue-induced ↑ reward ↓ DMN BOLD	N/A	N/A
Jansen et al. (105)	38 (20,18)	R.dlPFC	10	110	1 1 day	3,000	N/A	VAS	FC	N/A	↑ fcMRI of frontal pole	S	N
Mishra et al. (92)	20 (10 L, 10 R)	L.dlPFC R.dlPFC	10	110	10 10 days	1,000	N/A	ACQ	N/A	N/A	↓ in craving both left and right stimulation	D	N/A
Rapinesi et al. (101)	11	L.dlPFC	18	120	20 4 weeks	1,980	N/A	OCDS	N/A	N/A	↓ in craving/dep sx sustained at 6-months	N/A	N/A
Herremans et al. (116)	19	R.dlPFC	20	110	14 3 days	1,560	1-mo relapse	N/A	BOLD	N/A	68% relapse at 1mo ↓ dACC BOLD abstainers ↑ dACC BOLD relapsers	S	N
Qiao et al. (106)	38 (18,20)	R.dlPFC	10	80	4 5 days	800	N/A	N/A	MRS	HVLT, BVMT	↑ memory ↑ NAA/Cr and Cho/Cr	D	N
Del Felice et al. (117)	17 (8,9)	L.dlPFC	10	100	4 2 weeks	1,000	N/A	VAS	EEG	Stroop, Go-NoGo	No change in craving ↑ Stroop/Go-NoGo ↓ EEG/dep sx	S	N
Addolorato et al. (118)	11 (5,6)	L.dlPFC R.dlPFC	10	100	12 4 weeks	1,000	TLFB	OCDS	SPECTDAT	N/A	No diff in craving/dep sx ↓ in STAI-Y, DAT ↑ # of abstinent days,	D	Y
Hanlon et al. (95)	50 (25 coc, 25 alc)	vmPFC	5	110	6 1 day	3,600	N/A	N/A	BOLD	N/A	Alcohol: ↓ BOLD mPFC, AIns, MTG, and parahippocampal gyrus	S	Y
Hanlon et al. (96)	49 (25 coc, 24 alc)	vmPFC	5	110	6 1 day	3,600	N/A	VAS	BOLD	N/A	Alcohol: ↓ BOLD OFC, insula, and lateral sensorimotor cortex	S	Y
Kearney-Ramos et al. (97)	49 (25 coc, 24 alc)	vmPFC	5	110	6 1 day	3,600	N/A	VAS	FC	N/A	Alcohol: No diff in craving ↓ cue-related fcMRI of reward regions	S	Y
McNeill et al. (119)	20 (w/in- design)	R.dlPFC	50	80	1 1 day	600	drinking	N/A	N/A	Stop-signal	↓ inhibitory control ↑ alcohol consumption	N/A	N
Wu et al. (120)	51 (22, 29)	R.dlPFC	20	110	153 days	1,560	1-mo relapse	N/A	GMV	N/A	↓ GMV in relapsers, No change in TMS GMV but baseline predicted relapse	N/A	N/A
Perini et al. (121)	56 (29,27)	Bi-Insula	10	120	15 3 weeks	1,500	No TLFB	AUQ, PACS	BOLD	N/A	No diff in craving, drinking measures, fMRI	D	Y
Harel et al. (98)	51	mPFC ACC	10	100	15 3 weeks 5: 3mo fu	3,000	TLFB	PACS	FC	AUDIT ADS	↓ craving, ↓ % heavy drinking ↓rsFC dACC to caudate nucleus ↓FC mPFC to subgenual ACC.	D	Y

*Search terms included: alcohol use disorder and transcranial magnetic stimulation between 2010 and 2021. A review of the resulting articles was conducted by CBP and excluded position papers and reviews*.

### Other Considerations Moving Forward

**Figure 1:**
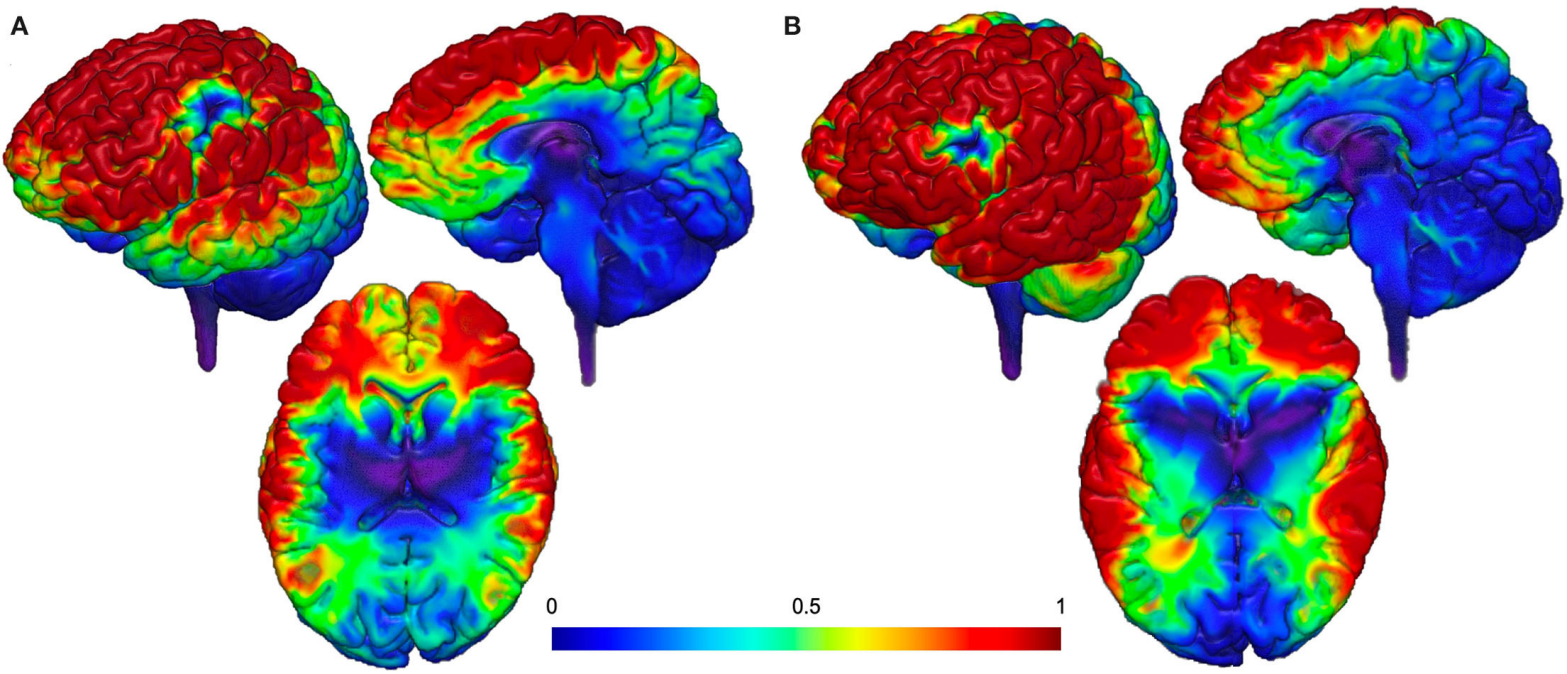
Proposed theoretical electrical field models (113). **(A)** Targeting dACC stimulation and **(B)** targeting AIns stimulation. Both models demonstrate additional delivery of a strong electrical field to the superficial cortical areas between the TMS coil and the deeper brain target.

As described above, the SN nodes are likely promising brain-based targets for therapeutic intervention, particularly by utilizing unique forms of TMS as tools to modulate deeper brain structures. However, one of the constraints of TMS is that current technology cannot reach the insula or dACC without also delivering a strong electric field to the superficial cortical areas between the TMS coil and the deeper brain target (as shown in Figure 1). To move the field forward, it is important to think creatively about non-invasive brain stimulation options that may allow us to selectively activate core SN nodes without simultaneously activating off-target cortical regions.

There are several complementary non-invasive approaches that may be useful for the field to consider as it evaluates the SN as a fruitful target for AUD treatment. One possibility is to use temporally interfering electric fields non-invasively applied at multiple cortical locations simultaneously. Grossman and colleagues recently demonstrated that by exploiting the inherent sensitivity of neural populations to varying frequencies, it is possible to selectively stimulate the mouse hippocampus. It remains unclear if this would yield similar results in humans.

Finally, while our focus has been on deep TMS (dTMS) potential, it is also possible to modulate these brain regions through a targeted cortical area with strong afferent projections to the cingulate or insula. This “cortical window” approach relies on the known ability of TMS to modulate areas monosynaptically connected to the area targeted by the electric field. This simple principle is evident by the basic generation of a motor evoked potential in the hand following stimulation of the primary motor cortex (a 2-synapse network). For example, active TMS applied to the frontal pole can change functional connectivity with the insula and cingulate cortex, when compared with sham TMS (96, 97). These are just a few considerations that the field may find fruitful when searching for a strategy to non-invasively modulate the SN nodes.

## Conclusion/Discussion: Potential Developments for the Field

Alcohol use disorder is highly prevalent, devastating, and notoriously difficult to effectively treat, as evidenced by the nearly two-thirds relapse rate within 6 months of treatment (123–125). One potential limitation of existing psychosocial and pharmaceutical interventions is that they modify behaviors more globally with indirect effects on the brain. Non-invasive neuromodulation techniques are showing promise toward the aim of modifying specific and selective neural targets related to AUD. However, device-based interventions to date for AUD have focused on superficial cortical stimulation, with most outcomes being related to craving. In contrast, preclinical and clinical studies suggest that deeper nodes within the SN could be promising targets, particularly the AIns and the dACC. Deep rTMS (dTMS) is one type of neuromodulation technique, utilizing an H-coil design (currently FDA-cleared for OCD and smoking cessation) that can potentially reach the AIns and dACC (126). However, it remains unclear if these targets are modifiable in AUD and which SN node (AIns or dACC or both) would have a greater impact on SN function, and importantly on reducing relapse risk post-treatment. Several lines of evidence support the SN as a promising future target for neuromodulation to impact treatment outcomes for AUD, and this warrants further investigation.

## Author Contributions

CP: conceptualization, resources, writing—original draft, supervision, and funding acquisition. L-TT: writing—original draft and writing—review and editing. DM: writing—review and editing and visualization. HA-D: literature review and writing—review and editing. CH, LW, FK, BK, TD, and JY: writing—review and editing. MM: conceptualization, resources, writing—original draft, and supervision. All authors contributed to the article and approved the submitted version.

## Funding

This research is based upon work funded by the Department of Veteran's Affairs (VA), Veteran's Health Administration, Office of Research and Development, Clinical Science Research and Development (CSR&D) Career Development Award (CDA-2), Grant Number IK2 CX001356 (PI: Padula), National Clinical rTMS Program funding from the Office of Mental Health and Suicide Prevention, and the Sierra Pacific VISN 21 Mental Illness Research, Education, and Clinical Center (MIRECC).

## Conflict of Interest

The authors declare that the research was conducted in the absence of any commercial or financial relationships that could be construed as a potential conflict of interest.

## Publisher's Note

All claims expressed in this article are solely those of the authors and do not necessarily represent those of their affiliated organizations, or those of the publisher, the editors and the reviewers. Any product that may be evaluated in this article, or claim that may be made by its manufacturer, is not guaranteed or endorsed by the publisher.

## References

[1] CESAR. Alcohol Reported as Primary Substance of Abuse in 62% of Veterans' Treatment Admissions. College Park, MD (2012). 2021:46. Available online at: http://db.cesar.umd.edu/cesar/cesarfax/vol21/21-46.pdf

[2] SAMHSA. National Household Survey on Drug Abuse: Main Findings 1998. (2010). p. 1–296.

[3] Spillane S Shiels MS Best AF Haozous EA Withrow DR Chen Y. Trends in alcohol-induced deaths in the United States, 2000–2016. JAMA Netw Open. (2020) 3:e1921451. 10.1001/jamanetworkopen.2019.21451 32083687 PMC7043198

[4] Durazzo TC Meyerhoff DJ. Psychiatric, demographic, and brain morphological predictors of relapse after treatment for an alcohol use disorder. Alcohol Clin Exp Res. (2017) 41:107–16. 10.1111/acer.13267 27883214 PMC6193554

[5] Durazzo TC Tosun D Buckley S Gazdzinski S Mon A Fryer SL. Cortical thickness, surface area, and volume of the brain reward system in alcohol dependence: relationships to relapse and extended abstinence. Alcohol Clin Exp Res. (2011) 35:1187–200. 10.1111/j.1530-0277.2011.01452.x 21410483 PMC3097308

[6] Durazzo TC Gazdzinski S Yeh P-H Meyerhoff DJ. Combined neuroimaging, neurocognitive and psychiatric factors to predict alcohol consumption following treatment for alcohol dependence. Alcohol Alcohol. (2008) 43:683–91. 10.1093/alcalc/agn078 18818189 PMC2720770

[7] Bouchery EE Harwood HJ Sacks JJ Simon CJ Brewer RD. Economic costs of excessive alcohol consumption in the U.S., 2006. Am J Prev Med. (2011) 41:516–24. 10.1016/j.amepre.2011.06.045 22011424

[8] Meyerhoff DJ Durazzo TC. Predicting relapse and treatment outcome in alcohol use disorders: time for an integrative biopsychosocial approach. Alcohol Clin Exp Res. (2010) 34:289–90.

[9] Koob GF Le Moal M. Drug abuse: hedonic homeostatic dysregulation. Science. (1997) 278:52–8. 10.1126/science.278.5335.52 9311926

[10] Koob GF Volkow ND. Neurocircuitry of Addiction. Neuropsychopharmacol. (2009) 35:217–238. 10.1038/npp.2009.110 19710631 PMC2805560

[11] Sinha R. Chronic stress, drug use, and vulnerability to addiction. Ann N Y Acad Sci. (2008) 1141:105. 10.1196/annals.1441.030 18991954 PMC2732004

[12] Seo D Sinha R. The neurobiology of alcohol craving and relapse. Handb Clin Neurol. (2014) 125:355–68. 10.1016/B978-0-444-62619-6.00021-5 25307585

[13] Dunlop K Hanlon CA Downar J. Noninvasive brain stimulation treatments for addiction and major depression. Ann N Y Acad Sci. (2017) 1394:31–54. 10.1111/nyas.12985 26849183 PMC5434820

[14] Seeley WW Menon V Schatzberg AF Keller J Glover GH Kenna H. Dissociable intrinsic connectivity networks for salience processing and executive control. J Neurosci. (2007) 27:2349. 10.1523/JNEUROSCI.5587-06.2007 17329432 PMC2680293

[15] Lindquist KA Barrett LF. A functional architecture of the human brain: emerging insights from the science of emotion. Trends Cogn Sci. (2012) 16:533. 10.1016/j.tics.2012.09.005 23036719 PMC3482298

[16] Oosterwijk S Lindquist KA Anderson E Dautoff R Moriguchi Y Barrett LF. States of mind: emotions, body feelings, and thoughts share distributed neural networks. Neuroimage. (2012) 62:2110–28. 10.1016/j.neuroimage.2012.05.079 22677148 PMC3453527

[17] Peters SK Dunlop K Downar J. Cortico-striatal-thalamic loop circuits of the salience network: a central pathway in psychiatric disease and treatment. Front Syst Neurosci. (2016) 10:104. 10.3389/fnsys.2016.00104 28082874 PMC5187454

[18] Williams LM. Precision psychiatry: a neural circuit taxonomy for depression and anxiety. Lancet Psychiatry. (2016) 3:472–80. 10.1016/S2215-0366(15)00579-9 27150382 PMC4922884

[19] Menon V. Salience network. In: Arthur W Toga E editors. Brain Mapping: An Encyclopedic Reference. Academic Press: Elsevier (2015) p.597–611.

[20] Sylvester CM Corbetta M Raichle ME Rodebaugh TL Schlaggar BL Sheline YI. Functional network dysfunction in anxiety and anxiety disorders. Trends Neurosci. (2012) 35:527–35. 10.1016/j.tins.2012.04.012 22658924 PMC3432139

[21] Tsai PJ Keeley RJ Carmack SA Vendruscolo JCM Lu H Gu H. Converging structural and functional evidence for a rat salience network. Biol Psychiatry. (2020) 88:867–78. 10.1016/j.biopsych.2020.06.023 32981657

[22] Feinstein JS Stein MB Paulus MP. Anterior insula reactivity during certain decisions is associated with neuroticism. Soc Cogn Affect Neurosci. (2006) 1:136. 10.1093/scan/nsl016 18985124 PMC2555442

[23] Naqvi NH Bechara A. The hidden island of addiction: the insula. Trends Neurosci. (2009) 32:56–67. 10.1016/j.tins.2008.09.009 18986715 PMC3698860

[24] Davis B Hasson U. Predictability of what or where reduces brain activity, but a bottleneck occurs when both are predictable. Neuroimage. (2018) 167:224–36. 10.1016/j.neuroimage.2016.06.001 27263508

[25] Lamichhane B Adhikari BM Dhamala M. Salience network activity in perceptual decisions. Brain Connect. (2016) 6:558–71. 10.1089/brain.2015.0392 27177981

[26] Ibrahim C Rubin-Kahana DS Pushparaj A Musiol M Blumberger DM Daskalakis ZJ. The insula: a brain stimulation target for the treatment of addiction. Front Pharmacol. (2019) 10:720. 10.3389/fphar.2019.00720 31312138 PMC6614510

[27] Craig AD. How do you feel? Interoception: the sense of the physiological condition of the body. Nat Rev Neurosci. (2002) 3:655–66. 10.1038/nrn894 12154366

[28] Naqvi S Zhu C Farre G Ramessar K Bassie L Breitenbach J. Transgenic multivitamin corn through biofortification of endosperm with three vitamins representing three distinct metabolic pathways. Proc Natl Acad Sci. (2009) 106:7762–7. 10.1073/pnas.0901412106 19416835 PMC2683132

[29] Bushara KO Grafman J Hallett M. Neural correlates of auditory–visual stimulus onset asynchrony detection. J Neurosci. (2001) 21:300–4. 10.1523/JNEUROSCI.21-01-00300.2001 11150347 PMC6762435

[30] Chang LJ Yarkoni T Khaw MW Sanfey AG. Decoding the role of the insula in human cognition: functional parcellation and large-scale reverse inference. Cereb Cortex. (2013) 23:739–49. 10.1093/cercor/bhs065 22437053 PMC3563343

[31] Ho TC Brown S Serences JT. Domain general mechanisms of perceptual decision making in human cortex. J Neurosci. (2009) 29:8675–87. 10.1523/JNEUROSCI.5984-08.2009 19587274 PMC2719543

[32] Lewis JW Beauchamp MS Deyoe EA. A comparison of visual and auditory motion processing in human cerebral cortex. Cereb Cortex. (2000) 10:873–88. 10.1093/cercor/10.9.873 10982748

[33] Sterzer P Kleinschmidt A. Anterior insula activations in perceptual paradigms: often observed but barely understood. Brain Struct Funct. (2010) 214:611–22. 10.1007/s00429-010-0252-2 20512379

[34] Goulden N Khusnulina A Davis NJ Bracewell RM Bokde AL McNulty JP. The salience network is responsible for switching between the default mode network and the central executive network: replication from DCM. Neuroimage. (2014) 99:180–90. 10.1016/j.neuroimage.2014.05.052 24862074

[35] Menon V. Large-scale brain networks and psychopathology: a unifying triple network model. Trends Cogn Sci. (2011) 15:483–506. 10.1016/j.tics.2011.08.003 21908230

[36] Menon V Uddin LQ. Saliency, switching, attention and control: a network model of insula function. Brain Struct Funct. (2010) 214:655–67. 10.1007/s00429-010-0262-0 20512370 PMC2899886

[37] Sridharan D Levitin DJ Menon V. A critical role for the right fronto-insular cortex in switching between central-executive and default-mode networks (2008). Available online at: www.pnas.org/cgi/content/full/ (accessed December 6, 2021). 10.1073/pnas.0800005105 PMC2527952 18723676

[38] Devinsky O Morrell MJ Vogt BA. Contributions of anterior cingulate cortex to behaviour. Brain. (1995) 118:279–306. 10.1093/brain/118.1.279 7895011

[39] Dosenbach NUF Fair DA Miezin FM Cohen AL Wenger KK Dosenbach RAT. Distinct brain networks for adaptive and stable task control in humans. Proc Natl Acad Sci. (2007) 104:11073–8. 10.1073/pnas.0704320104 17576922 PMC1904171

[40] Medford N Critchley HD. Conjoint activity of anterior insular and anterior cingulate cortex: awareness and response. Brain Struct Funct. (2010) 214:535–49. 10.1007/s00429-010-0265-x 20512367 PMC2886906

[41] Zysset S Wendt CS Volz KG Neumann J Huber O von Cramon DY. The neural implementation of multi-attribute decision making: a parametric fMRI study with human subjects. Neuroimage. (2006) 31:1380–8. 10.1016/j.neuroimage.2006.01.017 16513368

[42] Bush G Vogt BA Holmes J Dale AM Greve D Jenike MA. Dorsal anterior cingulate cortex: a role in reward-based decision making. Proc Natl Acad Sci. (2002) 99:523–8. 10.1073/pnas.012470999 11756669 PMC117593

[43] Paus T. Primate anterior cingulate cortex: where motor control, drive and cognition interface. Nat Rev Neurosci. (2001) 2:417–24. 10.1038/35077500 11389475

[44] Picard N Strick PL. Motor areas of the medial wall: a review of their location and functional activation. Cereb Cortex. (1996) 6:342–53. 10.1093/cercor/6.3.342 8670662

[45] Haber S. Corticostriatal circuitry. Dialogues Clin Neurosci. (2016) 18:7–21. 10.31887/DCNS.2016.18.1/shaber 27069376 PMC4826773

[46] Sha Z Wager TD Mechelli A He Y. Common dysfunction of large-scale neurocognitive networks across psychiatric disorders. Biol Psychiatry. (2019) 85:379–88. 10.1016/j.biopsych.2018.11.011 30612699

[47] Goodkind M Eickhoff SB Oathes DJ Jiang Y Chang A Jones-Hagata LB. Identification of a common neurobiological substrate for mental illness. JAMA psychiatry. (2015) 72:305–15. 10.1001/jamapsychiatry.2014.2206 25651064 PMC4791058

[48] McTeague LM Huemer J Carreon DM Jiang Y Eickhoff SB Etkin A. Identification of common neural circuit disruptions in cognitive control across psychiatric disorders. Am J Psychiatry. (2017) 174:676–85. 10.1176/appi.ajp.2017.16040400 28320224 PMC5543416

[49] Schacht JP Anton RF Myrick H. Functional neuroimaging studies of alcohol cue reactivity: a quantitative meta-analysis and systematic review. Addict Biol. (2013) 18:121–33. 10.1111/j.1369-1600.2012.00464.x 22574861 PMC3419322

[50] Wiers CE Stelzel C Gladwin TE Park SQ Pawelczack S Gawron CK. Effects of cognitivebias modification training on neural alcohol cue reactivity in alcohol dependence. Am J Psychiatry. (2015) 172:335–43. 10.1176/appi.ajp.2014.13111495 25526597

[51] Yang H Devous MD Briggs RW Spence JS Xiao H Kreyling N. Altered neural processing of threat in alcohol-dependent men. Alcohol Clin Exp Res. (2013) 37:2029–38. 10.1111/acer.12187 23888999 PMC4038904

[52] Salloum JB Ramchandani VA Bodurka J Rawlings R Momenan R George D. Blunted rostral anterior cingulate response during a simplified decoding task of negative emotional facial expressions in alcoholic patients. Alcohol Clin Exp Res. (2007) 31:1490–504. 10.1111/j.1530-0277.2007.00447.x 17624997

[53] Padula CB Anthenelli RM Eliassen JC Nelson E Lisdahl KM. Gender effects in alcohol dependence: an fMRI pilot study examining affective processing. Alcohol Clin Exp Res. (2015) 39:272–81. 10.1111/acer.12626 25684049 PMC5303611

[54] Zakiniaeiz Y Scheinost D Seo D Sinha R Constable RT. Cingulate cortex functional connectivity predicts future relapse in alcohol dependent individuals. NeuroImage Clin. (2017) 13:181–7. 10.1016/j.nicl.2016.10.019 27981033 PMC5144743

[55] Charlet K Schlagenhauf F Richter A Naundorf K Dornhof L Weinfurtner CEJ. Neural activation during processing of aversive faces predicts treatment outcome in alcoholism. Addict Biol. (2014) 19:439–51. 10.1111/adb.12045 23469861

[56] Beck A Schlagenhauf F Wüstenberg T Hein J Kienast T Kahnt T. Ventral striatal activation during reward anticipation correlates with impulsivity in alcoholics. Biol Psychiatry. (2009) 66:734–42. 10.1016/j.biopsych.2009.04.035 19560123

[57] Luijten M Machielsen MWJ Veltman DJ Hester R de Haan L Franken IHA. Systematic review of ERP and fMRI studies investigating inhibitory control and error processing in people with substance dependence and behavioural addictions. J Psychiatry Neurosci. (2014) 39:149–69. 10.1503/jpn.130052 24359877 PMC3997601

[58] Morris LS Kundu P Baek K Irvine MA Mechelmans DJ Wood J. Jumping the gun: mapping neural correlates of waiting impulsivity and relevance across alcohol misuse. Biol Psychiatry. (2016) 79:499–507. 10.1016/j.biopsych.2015.06.009 26185010 PMC4764648

[59] Claus ED Kiehl KA Hutchison KE. Neural and behavioral mechanisms of impulsive choice in alcohol use disorder. Alcohol Clin Exp Res. (2011) 35:1209–19. 10.1111/j.1530-0277.2011.01455.x 21676001 PMC3117198

[60] Dennis LE Kohno M McCready HD Schwartz DL Schwartz B Lahna D. Neural correlates of reward magnitude and delay during a probabilistic delay discounting task in alcohol use disorder. Psychopharmacology. (2020) 237:263–78. 10.1007/s00213-019-05364-3 31673722 PMC6991625

[61] Koob GF Volkow ND. Neurobiology of addiction: a neurocircuitry analysis. Lancet Psychiat. (2016) 3:760–73. 10.1016/S2215-0366(16)00104-8 27475769 PMC6135092

[62] Voon V Grodin E Mandali A Morris L Doñamayor N Weidacker K. Addictions neuroimaging assessment (ANIA): towards an integrative framework for alcohol use disorder. Neurosci Biobehav Rev. (2020) 113:492–506. 10.1016/j.neubiorev.2020.04.004 32298710

[63] Zilverstand A Huang AS Alia-Klein N Goldstein RZ. Neuroimaging impaired response inhibition and salience attribution in human drug addiction: a systematic review. Neuron. (2018) 98:886–903. 10.1016/j.neuron.2018.03.048 29879391 PMC5995133

[64] Kravitz A V Tomasi D Leblanc KH Baler R Volkow ND Bonci A. Cortico-striatal circuits: Novel therapeutic targets for substance use disorders. Brain Res. (2015) 1628:186–98. 10.1016/j.brainres.2015.03.048 25863130 PMC9364041

[65] Galandra C Basso G Manera M Crespi C Giorgi I Vittadini G. Salience network structural integrity predicts executive impairment in alcohol use disorders. Sci Reports. (2018) 8:1–13. 10.1038/s41598-018-32828-x 30262893 PMC6160480

[66] Grodin EN Cortes CR Spagnolo PA Momenan R. Structural deficits in salience network regions are associated with increased impulsivity and compulsivity in alcohol dependence. Drug Alcohol Depend. (2017) 179:100–8. 10.1016/j.drugalcdep.2017.06.014 28763777 PMC11034794

[67] Naqvi NH Rudrauf D Damasion H Bechara A. Damage to the insula disrupts addiction to cigarette smoking. Science. (2007) 315:531–4. 10.1126/science.1135926 17255515 PMC3698854

[68] Suñer-Soler R Grau A Gras ME Font-Mayolas S Silva Y Dávalos A. Smoking cessation 1 year poststroke and damage to the insular cortex. Stroke. (2012) 43:131–6. 10.1161/STROKEAHA.111.630004 22052507

[69] Abdolahi A Williams G Benesch C Wang H Spitzer E Scott B. Smoking cessation behaviors three months following acute insular damage from stroke. Addict Behav. (2015) 51:24–30. 10.1016/j.addbeh.2015.07.001 26188468 PMC4558299

[70] Canessa N Basso G Carne I Poggi P Gianelli C. Increased decision latency in alcohol use disorder reflects altered resting-state synchrony in the anterior salience network. Sci Reports. (2021) 11:1–11. 10.1038/s41598-021-99211-1 34599268 PMC8486863

[71] Sullivan E V Müller-Oehring E Pitel A-L Chanraud S Shankaranarayanan A Alsop DC. Selective insular perfusion deficit contributes to compromised salience network connectivity in recovering alcoholic men. Biol Psychiatry. (2013) 74:547. 10.1016/j.biopsych.2013.02.026 23587427 PMC3766441

[72] Gorka S Phan K Childs E. Acute calming effects of alcohol are associated with disruption of the salience network. Addict Biol. (2018) 23:921–30. 10.1111/adb.12537 28791789

[73] Butcher TJ Chumin EJ West JD Dzemidzic M Yoder KK. Cerebral blood flow in the salience network of individuals with alcohol use disorder. Alcohol Alcohol. (2021). 10.1093/alcalc/agab062. [Epub ahead of print]. 34541599 PMC9613478

[74] Padula CB Simmons AN Matthews SC Robinson SK Tapert SF Schuckit MA. Alcohol attenuates activation in the bilateral anterior insula during an emotional processing task: a pilot study. Alcohol Alcohol. (2011) 46:547–52. 10.1093/alcalc/agr066 21665869 PMC3201697

[75] Paulus MP Schuckit MA Tapert SF Tolentino NJ Matthews SC Smith TL. High versus low level of response to alcohol: evidence of differential reactivity to emotional stimuli. Biol Psychiatry. (2012) 72:848–55. 10.1016/j.biopsych.2012.04.016 22608014 PMC3433640

[76] Durazzo TC Pathak V Gazdzinski S Mon A Meyerhoff DJ. Metabolite levels in the brain reward pathway discriminate those who remain abstinent from those who resume hazardous alcohol consumption after treatment for alcohol dependence. J Stud Alcohol Drugs. (2010) 71:278–89. 10.15288/jsad.2010.71.278 20230726 PMC2841738

[77] Kohno M Dennis LE McCready H Hoffman WF. Executive control and striatal resting-state network interact with risk factors to influence treatment outcomes in alcohol-use disorder. Front Psychiatry. (2017) 8:182. 10.3389/fpsyt.2017.00182 28993741 PMC5622290

[78] Camchong J Haynos AF Hendrickson T Fiecas MB Gilmore CS Mueller BA. Resting hypoconnectivity of theoretically defined addiction networks during early abstinence predicts subsequent relapse in alcohol use disorder. Cereb Cortex. (2021). 10.1093/cercor/bhab374. [Epub ahead of print]. 34671808 PMC9393062

[79] Ihssen N Cox WM Wiggett A Fadardi JS Linden DEJ. Differentiating heavy from light drinkers by neural responses to visual alcohol cues and other motivational stimuli. Cereb Cortex. (2011) 21:1408–15. 10.1093/cercor/bhq220 21045002

[80] World Health Organization. Atlas on substance use (?2010)?: resources for the prevention and treatment of substance use disorders. World Health Organization (2010). Available online at: https://www.who.int/publications/i/item/9789241500616 (accessed September 10, 2021).

[81] McClintock S Reti I Carpenter L McDonald W Dubin M Taylor S. Consensus recommendations for the clinical application of repetitive transcranial magnetic stimulation (rTMS) in the treatment of depression. J Clin Psychiatry. (2018) 79:35–48. 10.4088/JCP.16cs10905 28541649 PMC5846193

[82] Perera T George M Grammer G Anicak P Pascual-Leone A Wirecki T. The clinical TMS society consensus review and treatment recommendations for TMS therapy for major depressive disorder. Brain Stimul. (2016) 9:336–46. 10.1016/j.brs.2016.03.010 27090022 PMC5612370

[83] Rossi S Hallett M Rossini P Pascual-Leone A. Safety, ethical considerations, and application guidelines for the use of transcranial magnetic stimulation in clinical practice and research. Clin Neurophysiol. (2009) 120:2008–39. 10.1016/j.clinph.2009.08.016 19833552 PMC3260536

[84] Rossi S Antal A Bestmann S Bikson M Brewer C Brockmöller J. Safety and recommendations for TMS use in healthy subjects and patient populations, with updates on training, ethical and regulatory issues: expert Guidelines. Clin Neurophysiol. (2020) 132:269–306. 10.1016/j.clinph.2020.10.003 33243615 PMC9094636

[85] Wassermann E. Risk and safety of repetitive transcranial magnetic stimulation: report and suggested guidelines from the International Workshop on the Safety of Repetitive Transcranial Magnetic Stimulation, June 5-7, 1996. Electroencephalogr Clin Neurophysiol. (1998) 108:1–16. 10.1016/S0168-5597(97)00096-8 9474057

[86] O'Reardon J Solvason H Janicak P Sampson S Isenberg K Nahas Z. Efficacy and safety of transcranial magnetic stimulation in the acute treatment of major depression: a multisite randomized controlled trial. Biol Psychiatry. (2007) 62:1208–16. 10.1016/j.biopsych.2007.01.018 17573044

[87] Roth Y Tendler A Arikan MK Vidrine R Kent D Muir O. Real-world efficacy of deep TMS for obsessive-compulsive disorder: post-marketing data collected from twenty-two clinical sites. J Psychiatr Res. (2021) 137:667–72. 10.1016/j.jpsychires.2020.11.009 33183769

[88] Young JR Galla JT Appelbaum LG. Transcranial magnetic stimulation treatment for smoking cessation: an introduction for primary care clinicians. Am J Med. (2021) 134:1339–343. 10.1016/j.amjmed.2021.06.037 34407423 PMC8607981

[89] Carpenter L Aaronson S Hutton TM Mina M Pages K Verdoliva S. Comparison of clinical outcomes with two transcranial magnetic stimulation treatment protocols for major depressive disorder. Brain Stimul Basic, Transl Clin Res Neuromodulation. (2021) 14:173–80. 10.1016/j.brs.2020.12.003 33346068

[90] Blumberger DM Vila-Rodriguez F Thorpe KE Feffer K Noda Y Giacobbe P. Effectiveness of theta burst versus high-frequency repetitive transcranial magnetic stimulation in patients with depression (THREE-D): a randomised non-inferiority trial. Lancet. (2018) 391:1683–92. 10.1016/S0140-6736(18)30295-2 29726344

[91] Antonelli M Fattore L Sestito L Di Giuda D Diana M Addolorato G. Transcranial magnetic stimulation: a review about its efficacy in the treatment of alcohol, tobacco and cocaine addiction. Addict Behav. (2021) 114:106760. 10.1016/j.addbeh.2020.106760 33316590

[92] Mishra B Praharaj S Katshu M Sarkar S Nizamie S. Comparison of anticraving efficacy of right and left repetitive transcranial magnetic stimulation in alcohol dependence: a randomized double-blind study. J Neuropsychiatry Clin Neurosci. (2015) 27:e54–9. 10.1176/appi.neuropsych.13010013 25255169

[93] Mishra BR Nizamie SH Das B Praharaj SK. Efficacy of repetitive transcranial magnetic stimulation in alcohol dependence: a sham-controlled study. Addiction. (2010) 105:49–55. 10.1111/j.1360-0443.2009.02777.x 20078462

[94] Herremans SC Van Schuerbeek P De Raedt R Matthys F Buyl R De Mey J. The impact of accelerated right prefrontal high-frequency repetitive transcranial magnetic stimulation (rTMS) on cue-reactivity: an fMRI study on craving in recently detoxified alcohol-dependent patients. PLoS ONE. (2015) 10:e0136182. 10.1371/journal.pone.0136182 26295336 PMC4546410

[95] Hanlon C Dowdle L Anton R George M. Ventral medial prefrontal cortex theta burst stimulation decreases salience network activity in cocaine users and alcohol users. Brain Stimul. (2017) 10:P479. 10.1016/j.brs.2017.01.405 PMC5896018 28686990

[96] Hanlon CA Dowdle LT Correia B Mithoefer O Kearney-Ramos T Lench D. Left frontal pole theta burst stimulation decreases orbitofrontal and insula activity in cocaine users and alcohol users. Drug Alcohol Depend. (2017) 178:310–17. 10.1016/j.drugalcdep.2017.03.039 28686990 PMC5896018

[97] Kearney-Ramos TE Dowdle LT Lench DH Mithoefer OJ Devries WH George MS. Transdiagnostic effects of ventromedial prefrontal cortex transcranial magnetic stimulation on cue reactivity. Biol Psychiatry Cogn Neurosci Neuroimaging. (2018) 3:599–609. 10.1016/j.bpsc.2018.03.016 29776789 PMC6641556

[98] Harel M Perini I Kämpe R Alyagon U Shalev H Besser I. Repetitive transcranial magnetic stimulation in alcohol dependence: a randomized, double-blind, sham-controlled proof-of-concept trial targeting medial prefrontal and anterior cingulate cortex. Biol Psychiatry. (2021). 10.1016/j.biopsych.2021.11.020. [Epub ahead of print]. 35067356

[99] Ceccanti M Inghilleri M Attilia M Raccah R Fiore M Zangen A. Deep TMS on alcoholics: effects on cortisolemia and dopamine pathway modulation. A pilot study. Can J Physiol Pharmacol. (2015) 93:283–90. 10.1139/cjpp-2014-0188 25730614

[100] Girardi P Rapinesi C Chiarotti F Kotzalidis GD Piacentino D Serata D. Add-on deep transcranial magnetic stimulation (dTMS) in patients with dysthymic disorder comorbid with alcohol use disorder: A comparison with standard treatment. World J Biol Psychiatry. (2015) 16:66–73. 10.3109/15622975.2014.925583 25140585

[101] Rapinesi C Curto M Kotzalidis GD Del Casale A Serata D Ferri VR. Antidepressant effectiveness of deep transcranial magnetic stimulation (dTMS) in patients with major depressive disorder (MDD) with or without alcohol use disorders (AUDs): a 6-month, open label, follow-up study. J Affect Disord. (2015) 174:57–63. 10.1016/j.jad.2014.11.015 25484178

[102] Höppner J Broese T Wendler L Berger C Thome J. Repetitive transcranial magnetic stimulation (rTMS) for treatment of alcohol dependence. World J Biol Psychiatry. (2011) 12:57–62. 10.3109/15622975.2011.598383 21905997

[103] Herremans SC Baeken C Vanderbruggen N Vanderhasselt MA Zeeuws D Santermans L. No influence of one right-sided prefrontal HF-rTMS session on alcohol craving in recently detoxified alcohol-dependent patients: results of a naturalistic study. Drug Alcohol Depend. (2012) 120:209–13. 10.1016/j.drugalcdep.2011.07.021 21855234

[104] Herremans SC Vanderhasselt MA De Raedt R Baeken C. Reduced intra-individual reaction time variability during a go-nogo task in detoxified alcohol-dependent patients after one right-sided dorsolateral prefrontal HF-rTMS session. Alcohol Alcohol. (2013) 48:552–7. 10.1093/alcalc/agt054 23709633

[105] Jansen JM Wingen G van Brink W van den Goudriaan AE. Resting state connectivity in alcohol dependent patients and the effect of repetitive transcranial magnetic stimulation. Eur Neuropsychopharmacol. (2015) 25:2230–239. 10.1016/j.euroneuro.2015.09.019 26481907

[106] Qiao J Jin G Lei L Wang L Du Y Wang X. The positive effects of high-frequency right dorsolateral prefrontal cortex repetitive transcranial magnetic stimulation on memory, correlated with increases in brain metabolites detected by proton magnetic resonance spectroscopy in recently detoxified al. Neuropsychiatr Dis Treat. (2016) 12:2273–8. 10.2147/NDT.S106266 27695332 PMC5028171

[107] Tendler A Barnea Ygael N Roth Y Zangen A. Deep transcranial magnetic stimulation (dTMS) - beyond depression. Expert Rev Med Devices. (2016) 13:987–1000. 10.1080/17434440.2016.1233812 27601183

[108] BrainsWay Ltd. Deep TMS System For Short Term Smoking Cessation Model 104: Instructions for Use (2021). IFU-0204-00. Available online at: https://www.brainsway.com/ as a customer of the manufacturer

[109] BrainsWay Ltd. Deep TMS System For Obsessive Compulsive Disorder Model 104: Instructions for Use: Revision 2.0 (2020). Available online at: https://www.brainsway.com/ as a customer of the manufacturer.

[110] Bohning DE Pecheny AP Epstein CM Speer AM Vincent DJ Dannels W. Mapping transcranial magnetic stimulation (TMS) fields *in vivo* with MRI. Neuroreport. (1997) 8:2535–8. 10.1097/00001756-199707280-00023 9261822

[111] Maxwell JC. On physical lines of force. Philos Mag. (2010) 90:11–23. 10.1080/14786431003659180

[112] Pfefferbaum A Sullivan EV Mathalon DH Lim KO. Frontal lobe volume loss observed with magnetic resonance imaging in older chronic alcoholics. Alcohol Clin Exp Res. (1997) 21:521–9. 10.1111/j.1530-0277.1997.tb03798.x 9161613

[113] McCalley DM Hanlon CA. Regionally specific gray matter volume is lower in alcohol use disorder: Implications for noninvasive brain stimulation treatment. Alcohol Clin Exp Res. (2021) 45:1672–83. 10.1111/acer.14654 34120347 PMC8560006

[114] Maiti R Mishra BR Hota D. Effect of high-frequency transcranial magnetic stimulation on craving in substance use disorder: a meta-analysis. Phschiarty. (2016) 29:160–71. 10.1176/appi.neuropsych.16040065 27707195

[115] De Ridder D Vanneste S Kovacs S Sunaert S Dom G. Transient alcohol craving suppression by rTMS of dorsal anterior cingulate: an fMRI and LORETA EEG study. Neurosci Lett. (2011) 496:5–10. 10.1016/j.neulet.2011.03.074 21458537

[116] Herremans SC De Raedt R Van Schuerbeek P Marinazzo D Matthys F De Mey J. Accelerated HF-rTMS protocol has a rate-dependent effect on dACC activation in alcohol-dependent patients: an open-label feasibility study. Alcohol Clin Exp Res. (2016) 40:196–205. 10.1111/acer.12937 26727534

[117] Del Felice A Bellamoli E Formaggio E Manganotti P Masiero S Cuoghi G. Neurophysiological, psychological and behavioural correlates of rTMS treatment in alcohol dependence. Drug Alcohol Depend. (2016) 158:147–53. 10.1016/j.drugalcdep.2015.11.018 26679060

[118] Addolorato G Antonelli M Cocciolillo F Vassallo GA Tarli C Sestito L. Deep transcranial magnetic stimulation of the dorsolateral prefrontal cortex in alcohol use disorder patients: effects on dopamine transporter availability and alcohol intake. Eur Neuropsychopharmacol. (2017) 27:450–61. 10.1016/j.euroneuro.2017.03.008 28390775

[119] McNeill A Monk RL Qureshi AW Makris S Heim D. Continuous theta burst transcranial magnetic stimulation of the right dorsolateral prefrontal cortex impairs inhibitory control and increases alcohol consumption. Cogn Affect Behav Neurosci. (2018) 18:1198. 10.3758/s13415-018-0631-3 30132267 PMC6244710

[120] Wu GR Baeken C Van Schuerbeek P De Mey J Bi M Herremans SC. Accelerated repetitive transcranial magnetic stimulation does not influence grey matter volumes in regions related to alcohol relapse: an open-label exploratory study. Drug Alcohol Depend. (2018) 191:210–14. 10.1016/j.drugalcdep.2018.07.004 30142603

[121] Perini I Kämpe R Arlestig T Karlsson H Löfberg A Pietrzak M. Repetitive transcranial magnetic stimulation targeting the insular cortex for reduction of heavy drinking in treatment-seeking alcohol-dependent subjects: a randomized controlled trial. Neuropsychopharmacology. (2020) 45:842–50. 10.1038/s41386-019-0565-7 31711065 PMC7075882

[122] Gaznick N Bechara A Tranel D. Basal ganglia plus insula damage yields stronger disruption of smoking addiction than basal ganglia damage alone (2014). Available online at: https://academic.oup.com/ntr/article-abstract/16/4/445/1172533 (accessed September 10, 2021). 10.1093/ntr/ntt172 PMC3954424 24169814

[123] Witkiewitz K Marlatt GA. Modeling the complexity of post-treatment drinking: It's a rocky road to relapse. Clin Psychol Rev. (2007) 27:724–38. 10.1016/j.cpr.2007.01.002 17355897 PMC1995671

[124] Maisto S Clifford P Stout RL Davis CM. Moderate drinking in the first year after treatment as a predictor of three-year outcomes. J Stud Alcohol Drugs. (2007) 68:419–27. 10.15288/jsad.2007.68.419 17446982

[125] Maisto SA Clifford PR Stout RL Davis CM. Drinking in the year after treatment as a predictor of three-year drinking outcomes. J Stud Alcohol Drugs. (2006) 67:823–32. 10.15288/jsa.2006.67.823 17060998

[126] U.S. Food and Drug Administration. De Novo Classification Request for BrainsWay Deep Transcrainal Magnetic Stimulation System Regulatory Information FDA. DEN170078 (2017). Available online at: https://www.accessdata.fda.gov/cdrh_docs/reviews/DEN170078.pdf

